# Interleukin-6 Test Strip Combined With a Spectrum-Based Optical Reader for Early Recognition of COVID-19 Patients With Risk of Respiratory Failure

**DOI:** 10.3389/fbioe.2022.796996

**Published:** 2022-02-15

**Authors:** Yung-Chih Wang, Sheng-Wen Lin, I-Jen Wang, Chung-Yao Yang, Chitsung Hong, Jun-Ren Sun, Po-Hao Feng, Mei-Hui Lee, Ching-Fen Shen, Yi-Tzu Lee, Chao-Min Cheng

**Affiliations:** ^1^ Division of Infectious Diseases and Tropical Medicine, Department of Internal Medicine, Tri-Service General Hospital, National Defense Medical Center, Taipei, Taiwan; ^2^ Institute of Biomedical Engineering, National Tsing Hua University, Hsinchu, Taiwan; ^3^ Department of Pediatrics, Taipei Hospital, Ministry of Health and Welfare, New Taipei City, Taiwan; ^4^ College of Public Health, China Medical University, Taichung, Taiwan; ^5^ School of Medicine, National Yang Ming Chiao Tung University, Taipei, Taiwan; ^6^ Hygeia Touch Inc., Taipei, Taiwan; ^7^ Spectrochip Inc., Hsinchu, Taiwan; ^8^ Institute of Preventive Medicine, National Defense Medical Center, Taipei, Taiwan; ^9^ School of Medicine, College of Medicine, Taipei Medical University, Taipei, Taiwan; ^10^ Division of Pulmonary Medicine, Department of Internal Medicine, Shuang Ho Hospital, Taipei, Taiwan; ^11^ Division of Infectious Diseases, Department of Internal Medicine, Shuang Ho Hospital, Taipei, Taiwan; ^12^ Department of Pediatrics, National Cheng Kung University Hospital, College of Medicine, National Cheng Kung University, Tainan, Taiwan; ^13^ Department of Emergency Medicine, Taipei Veterans General Hospital, Taipei, Taiwan

**Keywords:** COVID-19, interleukin-6, point-of-care, respiratory failure, SARS-CoV-2

## Abstract

The COVID-19 pandemic has had a globally devastating impact. This highly contagious virus has significantly overburdened and undermined medical systems. While most infected patients experience only mild symptoms, those who are severely affect require urgent medical interventions and some develop acute respiratory failure and require mechanical ventilation. The broad and potentially deadly impact of infection underscores the critical need for early recognition, especially for those at risk for respiratory failure. Those who are severely impacted and at high risk for respiratory failure have been found to present high levels of serum cytokines, such as interleukin-6 (IL-6). Timely diagnosis and management of those at risk for respiratory failure is crucial. Measurement of IL-6 may provide a means for distinguishing such patients. Currently, most serum IL-6 detection relies on the use of laboratory-based conventional enzyme-linked immunosorbent assays. Although some rapid assays have been developed recently, they need to be conducted by specific technicians in central laboratory settings with advanced and expensive equipment. In this study, we propose an IL-6 test strip combined with a spectrum-based optical reader for early recognition of COVID-19-infected patients at imminent risk of acute respiratory failure requiring mechanical ventilator support. For our analyses, clinical demographic data and sera samples were obtained from three medical centers, and test strip specificity and detection performance were analyzed. This would help healthcare personnel stratify the risk of respiratory failure and provide prompt, and suitable management.

## Introduction

Severe Acute Respiratory Syndrome coronavirus 2 (SARS-CoV-2) causing Coronavirus disease 2019 (COVID-19) has spread to pandemic levels, as designated on 11 March 2020 by the World Health Organization (WHO), and has accounted for more than 233, 136, 147 confirmed cases and 4,771,408 deaths as of 30 September 2021 (WHO, 2021). While most patients presented without symptoms or with mild to moderate symptoms, including fever, cough, fatigue, myalgias, nausea, and diarrhea, some may develop respiratory failure (Kim et al., 2020; Wu et al., 2020; Zhou et al., 2020). The rapid progression toward acute respiratory failure was the primary cause of mortality with an estimated case-fatality rate of approximately 2.1% to date (Grasselli et al., 2020; Huang et al., 2020).

Cytokine release syndrome (CRS) has been thought to contribute to pathogenesis in severe COVID-19 cases (Fajgenbaum and June 2020; Moore and June 2020). Several biomarkers, such as procalcitonin (Liu et al., 2020; Khodeir et al., 2021), C-reactive protein (Liu et al., 2020; Khodeir et al., 2021), interleukin-6 (IL-6) (Jørgensen et al., 2020; Liu et al., 2020; Khodeir et al., 2021), and monocyte chemotactic protein-1 (MCP-1) (Chen et al., 2020; Jørgensen et al., 2020) have been found to be associated with prognosis or disease severity in patients with COVID-19. As an important mediator of CRS, IL-6 appears to be correlated to respiratory failure (Jørgensen et al., 2020; Leisman et al., 2020; Moore and June 2020), acute respiratory distress syndrome (Leisman et al., 2020; Moore and June 2020), and adverse clinical outcomes(Del Valle et al., 2020; Moore and June 2020). While patients experiencing CRS had worsening clinical outcomes (Fajgenbaum and June 2020; Mangalmurti and Hunter, 2020), the early recognition of this immunological status could assist timely identification of patients who will progress to acute respiratory failure. It is important to distinguish between those with urgent medical needs from those experiencing only mild symptoms to avoid overloading medical care systems.

The breadth and scope of infection during a pandemic urges the need for an easy-to-use, inexpensive diagnostic tool to facilitate early diagnosis for those who are at high risk of respiratory failure. Enzyme-linked immunosorbent assay (ELISA) is a frequently used, well-developed (Lequin, 2005), traditional tool for measuring IL-6 concentration in laboratories (Yin et al., 2017). However, ELISA protocol requires multiple procedures and is time-consuming (Lequin, 2005). A point-of-care (POC) diagnostic tool for detection of IL-6 could overcome the drawbacks of traditional ELISA, such as time consuming, and need to be performed with multiple steps. The POC diagnostic tool could provide the results within minutes with only 1 to 2 steps and helps clinicians perform early-stage diagnosis of patients at risk for respiratory failure. Here, we proposed the use of a rapid IL-6 test strip which contains a lateral flow immunoassay (LFA)-based kit accompanied by a handheld optical reader that, in combination, is well suited for recognizing acute respiratory failure among COVID-19 patients. The spectrum-based optical reader is a palm-size portable chip-based spectrometric device (Hung et al., 2021), based on innovative micro-grating technology applied for biomedical spectral analysis (Kita et al., 2018; Wang et al., 2020a). In this study, we aimed to use a combination of an LFA technique-based IL-6 test strip and a sensitivity-enhanced spectrometric reader for early recognition of COVID-19 patients who may develop respiratory failure. We believe that this device could be a useful diagnostic tool for early identification of COVID-19-infected patients at risk for respiratory failure.

## Materials and Methods

### Patient Enrollment and Specimen Collection

Blood samples were obtained from patients diagnosed as COVID-19 from April 2020 to June 2021 at three hospitals in Taiwan: Tri-Service General Hospital (TSGH), Shuang Ho Hospital (SHH), and Taipei Hospital (TH) of Ministry of Health and Welfare (MOHW) in Northern Taiwan. The study protocol was approved by the institutional review boards (IRBs) of each site (TSGH: IRB No. C202005067, SHH: TMU-JIRB No. N202004076, TH: TH-IRB-0020-0011). The positive COVID-19 diagnosis was confirmed by real-time polymerase chain reaction testing for SARS-CoV-2 from nasopharyngeal samples. We reviewed patient medical records to retrieve clinical information, including the diagnosis of pneumonia and acute respiratory failure with mechanical ventilation. The severity of the COVID-19 was calssified according to the Infectious Diseases Society of America (IDSA) guidelines (Bhimraj et al., 2020). Critical illness was defined as patients on mechanical ventilation or extracorporeal membrane oxygenation (ECMO). Severe illness is defined as patients with SpO2 ≤94% on room air, including those on supplementary oxygen.

Blood samples were obtained within 48 h after the diagnosis of COVID-19. Follow-up blood samples were obtained 2 weeks after admission. The blood collected from the patients were allowed to clot by leaving it undisturbed at room temperature for 15–30 min. The blood clots were removed by centrifuging at 1,000–2,000 g for 10 min and resulting supernatant is designated serum. The serum was stored at temperatures of –20°C for further experiments.

### IL-6 Measurement in Serum Samples From Patients

Serum IL-6 concentrations were determined using commercially available ELISA kit (D6050, R&D systems, United States). The ELISA protocol was performed according to the manufacturer’s recommendations.

### Lateral Flow Immunoassay-Based IL-6 Test Strip

**FIGURE 1:**
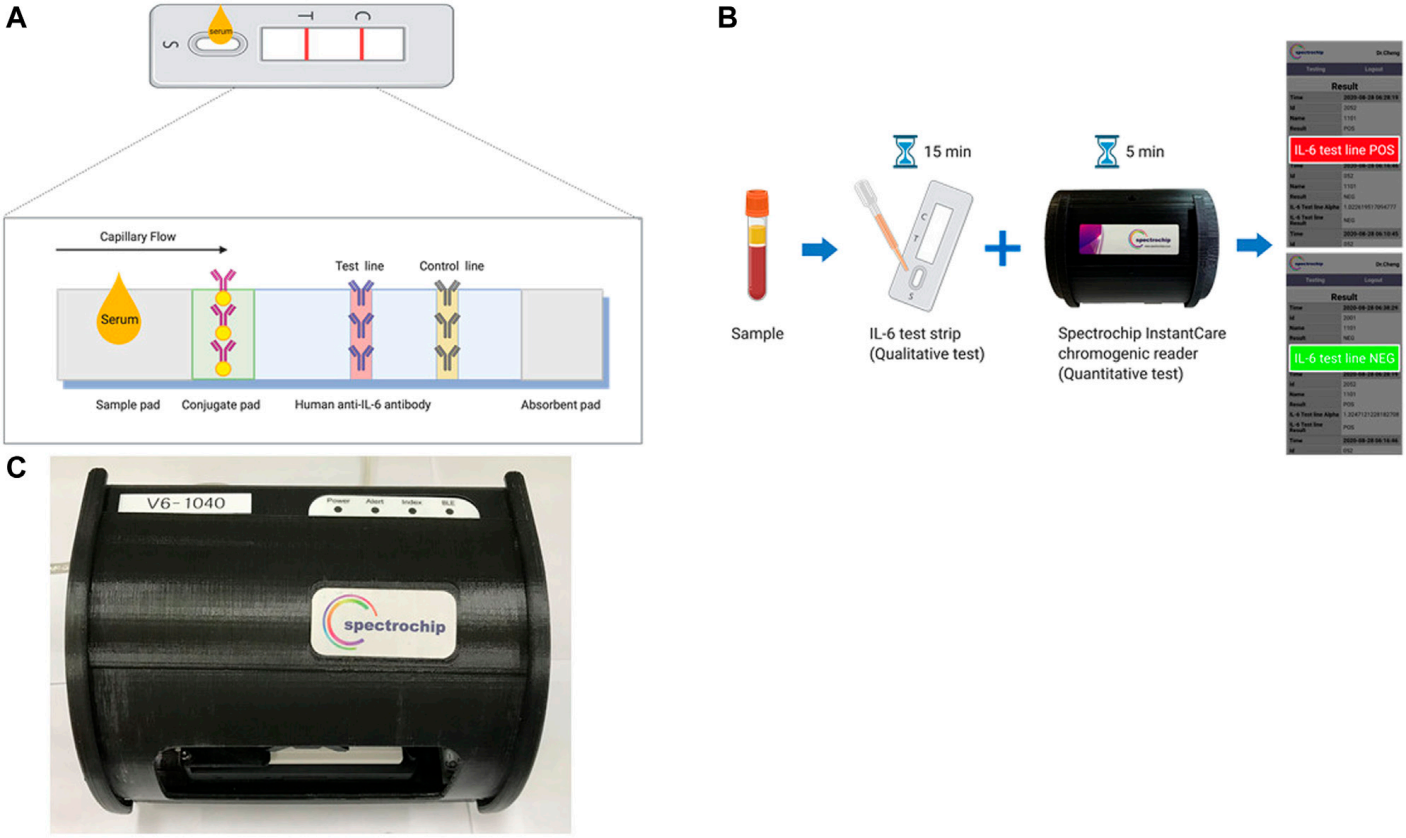
Interleukin 6 (IL-6) test strip and spectrum-based optical reader. **(A)** Schematic of the IL-6 lateral flow immunoassays. **(B)** The IL-6 test strip workflow couples with a spectrum-based optical reader. **(C)** The picture of the spectrum-based optical reader.

The test strip (created in cooperation with Taiwan Hygeia Touch Inc.) was developed based on the use of colloidal gold-conjugated anti-IL-6-antibody (ARG21446, arigo Biolaboratories Corp., Hsinchu, Taiwan) to detect human IL-6 (Figure 1A). An optimization test was performed to determine the most appropriate combination of IL-6 quantity and gold nanoparticle size for conjugation, which was based on colorimetric analysis of each combination. After the optimization test was performed, we used both a 15 nm-diameter gold colloid and an anti-IL-6 antibody (1 mg/ml) to prepare the IL-6 strip as previously described (Lin et al., 2021). The test strip was pre-coated with capture reagents by spraying it to create perpendicular lines of human anti-IL-6 antibodies (ARG21444, arigo Biolaboratories Corp., Hsinchu, Taiwan) conjugated with 15 nm-diameter gold colloids. This nitrocellulose strip was also coated with anti-mouse IgG monoclonal antibody (ARG21957, arigo Biolaboratories Corp., Hsinchu, Taiwan) at the test line (T line), as well as the same amount of the quality control antibody at the control line (C line). As the applied sample flowed down the strip, the human IL-6 antigen present in the sample formed an antigen complexed with the colloidal gold-labeled anti-IL-6-antibody. These complexes are then captured in the perpendicular T line of the test strip, where they were displayed as colored bands. To examine the IL-6 of the tested samples, a total of 100 μl of stored sera was pipetted into the sample application port and onto the test membrane, which was pretreated with a gold particle-conjugated monoclonal antibody specific for IL-6. The IL-6 in the sample bound to the gold particle-conjugated monoclonal anti-IL-6 antibodies in the membrane as the sample diffused through it and produce a visible colored line. Color intensity was directly proportional to the concentration of IL-6 in the sample. This color intensified during incubation. Surplus gold particles continued to diffuse over the test unit. Conjugate-specific antibodies printed as another line on the membrane (control line, C) captured the rest of the gold conjugate and a distinctly visible line developed during incubation. After incubation for 5 min, the color intensities were detected using the spectrum-based optical reader. The qualitative result was generated using a stored lot specific standard curve.

### Performance and Cross Reactivity of IL-6 Test Strip

Pre-determined concentrations (0, 0.1, 0.2, and 0.4 ng/ml) of IL-6 were tested using the IL-6 LFA test strips. Color intensity results on each test strip were analyzed using the spectrum-based optical reader, and the concentration of the IL-6 was determined based on a cutoff value for the spectral intensity. Potential test result interference for our test strips was evaluated by examining the impact of various endogenous substances (bilirubin, hemoglobin, intralipid, rheumatoid factor, IL-1α, IL-1β, IL-2, IL-3, IL-4, IL-8, and IL-9). These substances were examined for their impact on test interference, and tests were performed in triplicate.

### Reflectance Spectral Analysis

The spectrum analyzer (in cooperation with Taiwan SpectroChip Inc.; Taiwan FDA: MD(I)-008090 and US FDA: 3017810861) was equipped with a cassette designed for rapid optical IL-6 antibody reflectance spectrum detection (Figure 1B). This spectrum-based optical reader provided continuous-spectrum, high-resolution reflectance values when reading the result of test line (IL-6 line) via an optical module. The primary reflectance wavelengths detected by using this spectrum reader were 540 and 470 nm with the main reference wavelength being 650 nm (Hung et al., 2021).

The *α* value was calculated using the ratio of the spectra at the minimum reflectance and reference wavelengths: *α* = Reflectance (650 nm)/Reflectance (the minimum value in the range of 430–600 nm). A higher *α* value indicates a stronger reflection color intensity of the colloidal gold antibody-conjugated IL-6 complex, which indicates a higher antibody concentration (Lin et al., 2021).

### Statistical Analysis

The Spearman’s rank correlation coefficient and the Bland-Altman chart were used to check the correlation between our test strip/optical reader combination and ELISA. The sensitivity and specificity of the IL-6 test strips were also calculated.

## Results

### Clinical Demographic and Characteristics of Patients Enrolled in the Study

This study examined 50 blood samples obtained from 33 hospitalized patients with COVID-19. Among them, 10 (30.3%) patients had mild illness, 14 (42.42%) patients had COVID-19 pneumonia, and 9 patients (27.27%) had acute respiratory failure requiring intubation for respiratory support subsequent to Emergency Department presentation (Table 1). Because some of the study patients recovered well and experienced successful ventilator weaning, not all of them required a second blood sampling 2 weeks following diagnosis.

**TABLE 1:** Interleukin 6 (IL-6) concentrations and the severity of the COVID-19 patients.

Sample ID	IL-6 concentration (pg/ml)		Pneumonia	Mechanical ventilation
	ELISA	Spectrum-based optical reader quantitative estimate		
1	2.24	0	No	No
2	1	0	No	No
3	0.53	0	No	No
4	1.62	0	No	No
5	8.6	0	Yes	No
6	0	0	No	No
7	0	0	No	No
8	0	0	No	No
9	8.6	0	Yes	No
10	7.1	0	No	No
11	0	0	No	No
12	0.37	0	No	No
13	1.78	0	No	No
14	10.69	0	Yes	No
15	0.04	0	No	No
16	0	0	No	No
17	4.08	0	Yes	No
18	0	0	No	No
19	0.69	0	No	No
20	0.37	0	No	No
21	0.84	0	Yes	No
22	0	0	No	No
23	0.69	0	No	No
24	0	0	No	No
25	0	0	No	No
26	0.04	0	No	No
27	0.21	0	No	No
28	1.62	0	No	No
29	0	0	No	No
30	0	0	No	No
31	0.04	0	No	No
32	0	0	No	No
33	0.69	0	No	No
34	0	0	No	No
35	5.29	0	No	No
36	1.31	0	No	No
37	0.37	0	No	No
38	0.37	0	No	No
39	2.24	0	No	No
40	1	0	No	No
41	2.55	0	No	No
42	6,031.83	7,605	Yes	Yes
43	15.42	60.68	Yes	Yes
44	327.88	529.8	Yes	Yes
45	0	0	Yes	Yes
46	16.41	34.59	Yes	Yes
47	584.64	900.16	Yes	Yes
48	387.14	456.69	Yes	Yes
49	10.05	0	Yes	Yes
50	88.61	95.3	Yes	Yes

### The Performance of the IL-6 Test Strip

To evaluate the cross-reactivity of the test strip, a total of four endogenous compounds were examined for their impact on test interference up to the concentrations listed in Supplementary Table S1. No impact on results was observed from these tests, which were performed in triplicate. In addition, IL-6 (concentrations of approximately 0.1 ng/ml to 5 ng/ml) was mixed with seven other substances (Supplementary Table S2) to examine possible cross-reactivity. These substances demonstrated no influence on the IL-6 concentration results, indicating the high specificity of the IL-6 test strips.

### The Spectrum Analyzer System Provides High-Performance Spectral Analysis for the IL-6 Test Strip

**FIGURE 2:**
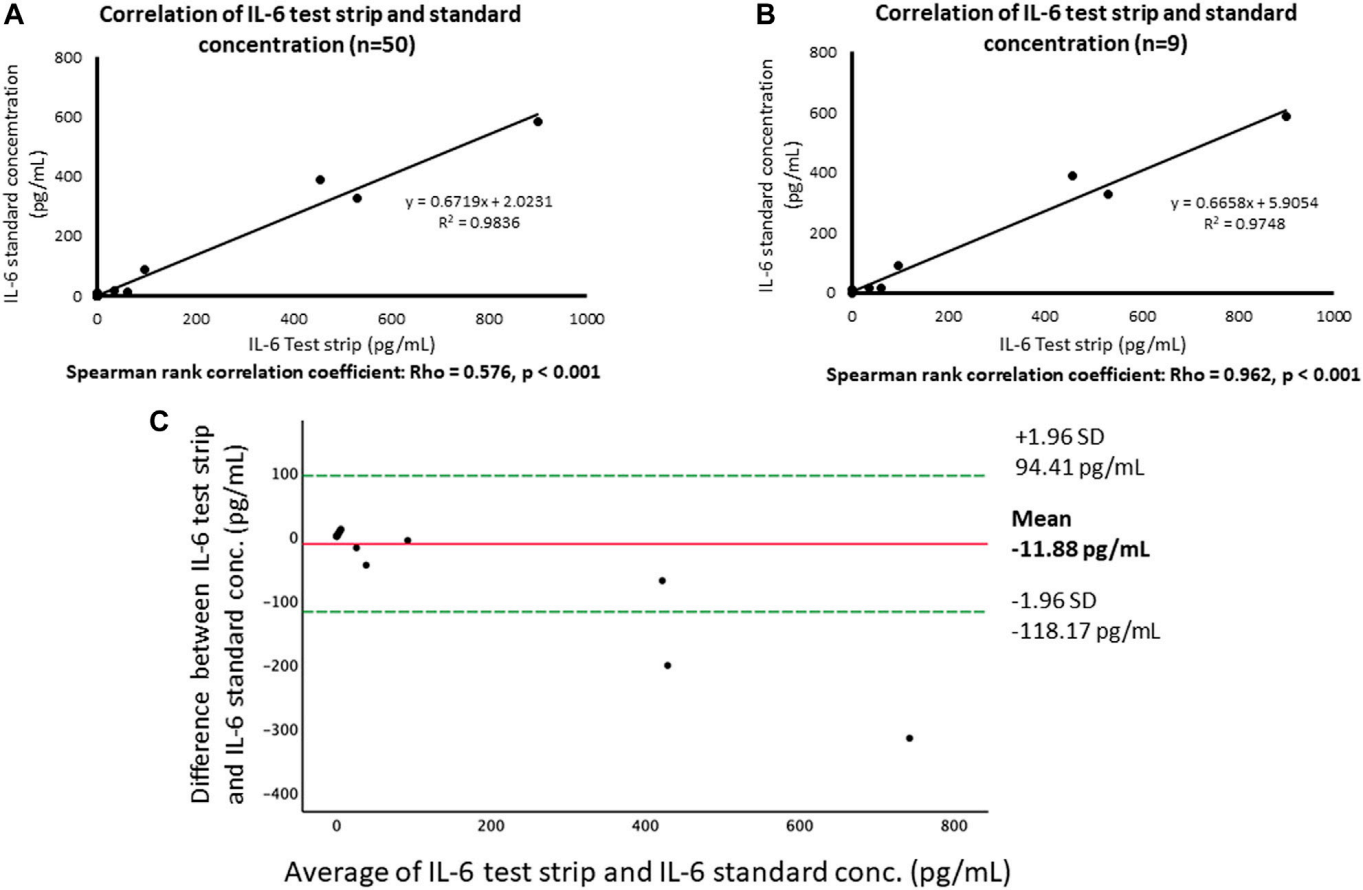
Comparison of our test strip and ELISA for IL-6 assays. **(A)** Correlation of IL-6 test strip and IL-6 standard concentration. **(B)** Correlation of IL-6 test strip and IL-6 standard concentration (severe). **(C)** Bland and Altman plot of log-transformed data. The differences between the IL-6 based on test strip and ELISA (log transformation) in relation to the mean of the two measurements (log transformation), n = 49. Green lines indicate the limits of agreement (±1.96 SD.).

Figure 2A shows that the correlation between the concentration of IL-6 test strip and ELISA were highly consistent, *R* ^2^ = 0.983. For patients requiring mechanical ventilation for acute respiratory failure (Figure 2B), the results of IL-6 test strip also exhibited a high correlation with ELISA results, *R* ^2^ = 0.974. In addition, in the spearman method, the correlation between the 50 blood samples tested and the correlation between the tested samples from those with mechanical ventilation are significantly different (*p* < 0.001), and the Rho is 0.576 and 0.962, respectively. The Bland-Altman plot shows the comparison of each difference between the two pairing methods with the measured average, as shown in Figure 2C. The performance of the device has been previously documented as providing a limit of detection and limit of quantification of 76.85 and 402.71 pg/ml, respectively (Lin et al., 2021).

### Recognition of Acute Respiratory Failure Requiring Mechanical Ventilation Using IL-6 Test Strip

Serum IL-6 concentration, presence of pneumonia, and the receipt of mechanical ventilation for acute respiratory failure are listed in Table 1. Seven of the nine patients with mechanical ventilation had an elevated IL-6 concentration higher than 76.85 pg/ml (range: 76.85–7605 pg/ml). Using a cutoff value of 76.85 pg/ml, the IL-6 test strip exhibited 100% sensitivity and 96% specificity for the recognition of mechanical ventilation (Supplementary Table S3). Samples from two patients (sample No. 45 and No. 49) who received mechanical ventilation showed undetectable serum IL-6.

## Discussion

The integration of a rapid, easy-to-use IL-6 test strip and a sensitivity-enhanced spectrometric reader makes this POC diagnostic device a promising tool for early recognition of patients who may develop respiratory failure requiring mechanical ventilation. The limit of detection was as low as 76.85 pg/ml and sensitive enough for clinical application as we previously mentioned (Lin et al., 2021).

IL-6 is an important cytokine that is rapidly induced over the course of infection (Ma et al., 2016) and could be used as a diagnostic aid to confirm infection in patients with systemic inflammatory response syndrome (Ma et al., 2016). It has previously been found to be associated with disease severity and poor outcome for patients with sepsis (Song and Kellum, 2005; Ventetuolo and Levy, 2008; Song et al., 2019). Elevated IL-6 is also known to be associated with CRS (Fajgenbaum and June 2020; Mangalmurti and Hunter, 2020) and induces a more severe disease and worse outcome for patients with COVID-19 (Jørgensen et al., 2020; Liu et al., 2020; Khodeir et al., 2021). It could, thus, be used as a marker to stratify those with risk for severe disease. A meta-analysis study including 37 studies showed that mean IL-6 concentration was 55.3 and 37.3 pg/ml in patients with critical and severe COVID-19, respectively. (Leisman et al., 2020), and 1,558.2 pg/ml in COVID-19 patients with hyperinflammatory acute respiratory distress syndrome (ARDS) (Leisman et al., 2020).

Three IL-6 diagnostic assays, Elecsys IL-6 (Roche Diagnostics, Basel, Switzerland) (Elecsys, 2021), Access IL-6 assay (Beckman Coulter, Inc., Brea, California, US), (Beckmancoulter, 2021), and ADVIA Centaur IL-6 assay (Siemens Healthcare Diagnostics, Inc., Erlangen, Germany) (Siemens Healthineers, 2021) were authorized for use by the US FDA by the end of April 2021. Each of these tests, in conjunction with clinical findings and the results of other laboratory testing, have been used to assist in identifying severe inflammatory response as a means of determining the risk of intubation with mechanical ventilation. However, these tests needed to be performed by well-trained technicians in a central laboratory with specific equipment. A POC diagnostic tool could be performed more easily and could shorten the time for diagnosis (Wang et al., 2020b). In this study, we demonstrated a novel IL-6 detection POC diagnostic device that could detect those at risk of respiratory failure. Using a cutoff level of 76.85 pg/ml, we successfully differentiated COVID-19 patients with acute respiratory failure from those without acute respiratory failure.

This study had several limitations. Although our test strip combined with a spectrum-based optical reader showed an excellent detection limit using the standard IL-6 compound, the sample size was small, and a further study including a larger sample size should be performed. Second, some patients received steroids or other immunosuppressants for COVID-19 treatment, which would influence serum IL-6 level. In addition, underlying comorbidities, such as malignancy and chronic lung disease, may have potentiated respiratory failure in those with COVID-19. Patients with these underlying diseases may develop respiratory failure even if they have low serum IL-6 levels.

In conclusion, this study showed that an IL-6 LFA test strip combined with a spectrometric reader could provide a promising POC diagnostic approach for early recognition of COVID-19 patients who are at risk for acute respiratory failure. This would facilitate clinical risk assessment and stratification for COVID-19 patients, and subsequently ease burdens currently placed on global healthcare systems.

## Data Availability

The raw data supporting the conclusions of this article will be made available by the authors, without undue reservation.
