# Supplementary material for: Interleukin-6 Test Strip Combined With a Spectrum-Based Optical Reader for Early Recognition of COVID-19 Patients With Risk of Respiratory Failure
Source: Front Bioeng Biotechnol. 2022 Feb 15;10:796996. doi: 10.3389/fbioe.2022.796996 (PMC8886233; doi:10.3389/fbioe.2022.796996)
Supplement: Supplementary file 1 [file DataSheet1.docx]

**Supplementary Table S1. Endogenous compounds used for cross reactivity test.**

| **Compounds/Substances** | **Concentration Tested** |
| --- | --- |
| Bilirubin | ≤ 40 mg/dL |
| Hemoglobin | ≤ 1000 mg/dL |
| Intralipid | ≤ 1500 mg/dL |
| Rheumatoid factors | ≤ 1200 IU/mL |

**Supplementary Table S2. Cytokines other than IL-6 used for cross reactivity test.**

| **Substances** | **Concentration Tested** |
| --- | --- |
| Interleukin-1α | 50 ng/mL |
| Interleukin-1β | 50 ng/mL |
| Interleukin-2 | 50 ng/mL |
| Interleukin-3 | 50 ng/mL |
| Interleukin-4 | 50 ng/mL |
| Interleukin-8 | 50 ng/mL |
| Interleukin-9 | 50 ng/mL |

**Supplementary table S3. Performance of the Interleukin-6 test strip.**

|  |  | Clinical outcomes | | |
| --- | --- | --- | --- | --- |
|  |  | Mechanical ventilation patients | No mechanical ventilation required | Total |
| IL-6 Test Kit | Positive | 8 | 1 | 9 |
|  | Negative | 0 | 24 | 24 |
|  | Total | 8 | 25 | 33 |
| Sensitivity | | 100 % |  |  |
| Specificity | | 96 %  (95 % CI: 87 % to 100 %) |  |  |
